# Supplementary material for: Resolving the full spectrum of human genome variation using Linked-Reads
Source: Genome Res. 2019 Apr;29(4):635–45. doi: 10.1101/gr.234443.118 (PMC6442396; doi:10.1101/gr.234443.118)
Supplement: Supplemental Material [file supp_gr.234443.118_Supplemental_longranger-2.2.zip › longranger-2.2.tar.bz2!/lr2.2-public/lib/stan/cmdstan/stan/doc/api/html/classstan_1_1mcmc_1_1base__nuts.html]

Stan: stan::mcmc::base\_nuts< M, P, H, I, BaseRNG > Class Template Reference


|  |  |
| --- | --- |
|  | Stan  2.5.0  probability, sampling & optimization |


- Main Page
- Related Pages
- Namespaces
- Classes
- Files

- Class List
- Class Index
- Class Hierarchy
- Class Members

All Classes Namespaces Files Functions Variables Typedefs Enumerations Enumerator Friends Macros Pages

- stan
- mcmc
- base\_nuts

Public Member Functions |
Public Attributes |
List of all members

stan::mcmc::base\_nuts< M, P, H, I, BaseRNG > Class Template Referenceabstract

`#include <base_nuts.hpp>`

Inheritance diagram for stan::mcmc::base\_nuts< M, P, H, I, BaseRNG >:

|  |  |
| --- | --- |
| Public Member Functions | |
|  | base\_nuts (M &m, BaseRNG &rng, std::ostream \*o, std::ostream \*e) |
|  | |
|  | ~base\_nuts () |
|  | |
| void | set\_max\_depth (const int d) |
|  | |
| void | set\_max\_delta (const double d) |
|  | |
| int | get\_max\_depth () |
|  | |
| double | get\_max\_delta () |
|  | |
| sample | transition (sample &init\_sample) |
|  | |
| void | write\_sampler\_param\_names (std::ostream &o) |
|  | |
| void | write\_sampler\_params (std::ostream &o) |
|  | |
| void | get\_sampler\_param\_names (std::vector< std::string > &names) |
|  | |
| void | get\_sampler\_params (std::vector< double > &values) |
|  | |
| virtual bool | compute\_criterion (ps\_point &start, P &finish, Eigen::VectorXd &rho)=0 |
|  | |
| int | build\_tree (int depth, Eigen::VectorXd &rho, ps\_point \*z\_init\_parent, ps\_point &z\_propose, nuts\_util &util) |
|  | |
| Public Member Functions inherited from stan::mcmc::base\_hmc< M, P, H, I, BaseRNG > | |
|  | base\_hmc (M &m, BaseRNG &rng, std::ostream \*o, std::ostream \*e) |
|  | |
| void | write\_sampler\_state (std::ostream \*o) |
|  | |
| void | get\_sampler\_diagnostic\_names (std::vector< std::string > &model\_names, std::vector< std::string > &names) |
|  | |
| void | get\_sampler\_diagnostics (std::vector< double > &values) |
|  | |
| void | seed (const Eigen::VectorXd &q) |
|  | |
| void | init\_stepsize () |
|  | |
| P & | z () |
|  | |
| virtual void | set\_nominal\_stepsize (const double e) |
|  | |
| double | get\_nominal\_stepsize () |
|  | |
| double | get\_current\_stepsize () |
|  | |
| virtual void | set\_stepsize\_jitter (const double j) |
|  | |
| double | get\_stepsize\_jitter () |
|  | |
| void | sample\_stepsize () |
|  | |
| Public Member Functions inherited from stan::mcmc::base\_mcmc | |
|  | base\_mcmc (std::ostream \*o, std::ostream \*e) |
|  | |
| virtual | ~base\_mcmc () |
|  | |
| std::string | name () |
|  | |

|  |  |
| --- | --- |
| Public Attributes | |
| int | depth\_ |
|  | |
| int | max\_depth\_ |
|  | |
| double | max\_delta\_ |
|  | |
| int | n\_leapfrog\_ |
|  | |
| int | n\_divergent\_ |
|  | |

|  |  |
| --- | --- |
| Additional Inherited Members | |
| Protected Attributes inherited from stan::mcmc::base\_hmc< M, P, H, I, BaseRNG > | |
| P | z\_ |
|  | |
| I< H< M, BaseRNG >, P > | integrator\_ |
|  | |
| H< M, BaseRNG > | hamiltonian\_ |
|  | |
| BaseRNG & | rand\_int\_ |
|  | |
| boost::uniform\_01< BaseRNG & > | rand\_uniform\_ |
|  | |
| double | nom\_epsilon\_ |
|  | |
| double | epsilon\_ |
|  | |
| double | epsilon\_jitter\_ |
|  | |
| Protected Attributes inherited from stan::mcmc::base\_mcmc | |
| std::string | name\_ |
|  | |
| std::ostream \* | out\_stream\_ |
|  | |
| std::ostream \* | err\_stream\_ |
|  | |

## Detailed Description

### template<class M, class P, template< class, class > class H, template< class, class > class I, class BaseRNG> class stan::mcmc::base\_nuts< M, P, H, I, BaseRNG >

Definition at line 32 of file base\_nuts.hpp.

## Constructor & Destructor Documentation

template<class M, class P, template< class, class > class H, template< class, class > class I, class BaseRNG>

|  |  |  |  |
| --- | --- | --- | --- |
| stan::mcmc::base\_nuts< M, P, H, I, BaseRNG >::base\_nuts | ( | M & | *m*, |
|  |  | BaseRNG & | *rng*, |
|  |  | std::ostream \* | *o*, |
|  |  | std::ostream \* | *e* |
|  | ) |  |  |

inline

Definition at line 37 of file base\_nuts.hpp.

template<class M, class P, template< class, class > class H, template< class, class > class I, class BaseRNG>

|  |  |  |  |  |
| --- | --- | --- | --- | --- |
| stan::mcmc::base\_nuts< M, P, H, I, BaseRNG >::~base\_nuts | ( |  | ) |  |

inline

Definition at line 42 of file base\_nuts.hpp.

## Member Function Documentation

template<class M, class P, template< class, class > class H, template< class, class > class I, class BaseRNG>

|  |  |  |  |
| --- | --- | --- | --- |
| int stan::mcmc::base\_nuts< M, P, H, I, BaseRNG >::build\_tree | ( | int | *depth*, |
|  |  | Eigen::VectorXd & | *rho*, |
|  |  | ps\_point \* | *z\_init\_parent*, |
|  |  | ps\_point & | *z\_propose*, |
|  |  | nuts\_util & | *util* |
|  | ) |  |  |

inline

Definition at line 181 of file base\_nuts.hpp.

template<class M, class P, template< class, class > class H, template< class, class > class I, class BaseRNG>

|  |  |  |  |
| --- | --- | --- | --- |
| virtual bool stan::mcmc::base\_nuts< M, P, H, I, BaseRNG >::compute\_criterion | ( | ps\_point & | *start*, |
|  |  | P & | *finish*, |
|  |  | Eigen::VectorXd & | *rho* |
|  | ) |  |  |

pure virtual

Implemented in stan::mcmc::dense\_e\_nuts< M, BaseRNG >, stan::mcmc::diag\_e\_nuts< M, BaseRNG >, and stan::mcmc::unit\_e\_nuts< M, BaseRNG >.

template<class M, class P, template< class, class > class H, template< class, class > class I, class BaseRNG>

|  |  |  |  |  |
| --- | --- | --- | --- | --- |
| double stan::mcmc::base\_nuts< M, P, H, I, BaseRNG >::get\_max\_delta | ( |  | ) |  |

inline

Definition at line 54 of file base\_nuts.hpp.

template<class M, class P, template< class, class > class H, template< class, class > class I, class BaseRNG>

|  |  |  |  |  |
| --- | --- | --- | --- | --- |
| int stan::mcmc::base\_nuts< M, P, H, I, BaseRNG >::get\_max\_depth | ( |  | ) |  |

inline

Definition at line 53 of file base\_nuts.hpp.

template<class M, class P, template< class, class > class H, template< class, class > class I, class BaseRNG>

|  |  |  |  |  |  |
| --- | --- | --- | --- | --- | --- |
| void stan::mcmc::base\_nuts< M, P, H, I, BaseRNG >::get\_sampler\_param\_names | ( | std::vector< std::string > & | *names* | ) |  |

inlinevirtual

Reimplemented from stan::mcmc::base\_mcmc.

Definition at line 164 of file base\_nuts.hpp.

template<class M, class P, template< class, class > class H, template< class, class > class I, class BaseRNG>

|  |  |  |  |  |  |
| --- | --- | --- | --- | --- | --- |
| void stan::mcmc::base\_nuts< M, P, H, I, BaseRNG >::get\_sampler\_params | ( | std::vector< double > & | *values* | ) |  |

inlinevirtual

Reimplemented from stan::mcmc::base\_mcmc.

Definition at line 171 of file base\_nuts.hpp.

template<class M, class P, template< class, class > class H, template< class, class > class I, class BaseRNG>

|  |  |  |  |  |  |
| --- | --- | --- | --- | --- | --- |
| void stan::mcmc::base\_nuts< M, P, H, I, BaseRNG >::set\_max\_delta | ( | const double | *d* | ) |  |

inline

Definition at line 49 of file base\_nuts.hpp.

template<class M, class P, template< class, class > class H, template< class, class > class I, class BaseRNG>

|  |  |  |  |  |  |
| --- | --- | --- | --- | --- | --- |
| void stan::mcmc::base\_nuts< M, P, H, I, BaseRNG >::set\_max\_depth | ( | const int | *d* | ) |  |

inline

Definition at line 44 of file base\_nuts.hpp.

template<class M, class P, template< class, class > class H, template< class, class > class I, class BaseRNG>

|  |  |  |  |  |  |
| --- | --- | --- | --- | --- | --- |
| sample stan::mcmc::base\_nuts< M, P, H, I, BaseRNG >::transition | ( | sample & | *init\_sample* | ) |  |

inlinevirtual

Implements stan::mcmc::base\_mcmc.

Definition at line 56 of file base\_nuts.hpp.

template<class M, class P, template< class, class > class H, template< class, class > class I, class BaseRNG>

|  |  |  |  |  |  |
| --- | --- | --- | --- | --- | --- |
| void stan::mcmc::base\_nuts< M, P, H, I, BaseRNG >::write\_sampler\_param\_names | ( | std::ostream & | *o* | ) |  |

inlinevirtual

Reimplemented from stan::mcmc::base\_mcmc.

Definition at line 155 of file base\_nuts.hpp.

template<class M, class P, template< class, class > class H, template< class, class > class I, class BaseRNG>

|  |  |  |  |  |  |
| --- | --- | --- | --- | --- | --- |
| void stan::mcmc::base\_nuts< M, P, H, I, BaseRNG >::write\_sampler\_params | ( | std::ostream & | *o* | ) |  |

inlinevirtual

Reimplemented from stan::mcmc::base\_mcmc.

Definition at line 159 of file base\_nuts.hpp.

## Member Data Documentation

template<class M, class P, template< class, class > class H, template< class, class > class I, class BaseRNG>

int stan::mcmc::base\_nuts< M, P, H, I, BaseRNG >::depth\_

Definition at line 247 of file base\_nuts.hpp.

template<class M, class P, template< class, class > class H, template< class, class > class I, class BaseRNG>

double stan::mcmc::base\_nuts< M, P, H, I, BaseRNG >::max\_delta\_

Definition at line 249 of file base\_nuts.hpp.

template<class M, class P, template< class, class > class H, template< class, class > class I, class BaseRNG>

int stan::mcmc::base\_nuts< M, P, H, I, BaseRNG >::max\_depth\_

Definition at line 248 of file base\_nuts.hpp.

template<class M, class P, template< class, class > class H, template< class, class > class I, class BaseRNG>

int stan::mcmc::base\_nuts< M, P, H, I, BaseRNG >::n\_divergent\_

Definition at line 252 of file base\_nuts.hpp.

template<class M, class P, template< class, class > class H, template< class, class > class I, class BaseRNG>

int stan::mcmc::base\_nuts< M, P, H, I, BaseRNG >::n\_leapfrog\_

Definition at line 251 of file base\_nuts.hpp.

---

The documentation for this class was generated from the following file:

- src/stan/mcmc/hmc/nuts/base\_nuts.hpp


---

[ Stan Home Page ]

*© 2011–2014,
Stan Development Team.*
